# A Comparison of a Traditional Clinical Experience to a Precepted Clinical Experience for Baccalaureate-Seeking Nursing Students in Their Second Semester

**DOI:** 10.1155/2012/276506

**Published:** 2012-04-22

**Authors:** Kristin Ownby, Renae Schumann, Linda Dune, David Kohne

**Affiliations:** The University of Texas Health Science Center School of Nursing at Houston, 6901 Bertner Avenue, Room 691, Houston, TX 77030, USA

## Abstract

The shortage of nursing faculty has contributed greatly to the nursing workforce shortage, with many schools turning away qualified applicants because there are not enough faculty to teach. Despite the faculty shortage, schools are required to admit more students to alleviate the nursing shortage. Clinical groups in which preceptors are responsible for student learning extend faculty resources. *Purpose.* To determine the effectiveness of an alternative clinical experience (preceptorship). *Methods.* quasi-experimental, randomized, longitudinal design. Students were randomized to either the traditional or precepted clinical group. The clinical experience was a total of 12 weeks. Groups were compared according to several variables including second semester exam scores, HESI scores, and quality and timeliness of clinical paperwork. *Sample.* Over a two-year period, seventy-one undergraduate nursing students in the second semester medical-surgical nursing course participated. 36 were randomized to the experimental group. The preceptors were baccalaureate-prepared nurses who have been practicing for at least one year. *Setting.* Two hospitals located in the Texas Medical Center. *Statistical Analysis.* Descriptive statistics and independent *t*-test. *Results.* There was no difference between the groups on the variables of interest. *Conclusion.* Students in the precepted clinical group perform as well as those in a traditional clinical group.

## 1. Introduction

A recent report published by the American Association of Colleges of Nursing (AACN) noted that over 67,000 qualified applicants were not accepted into baccalaureate and graduate nursing programs in the USA in 2010. The report also noted that almost two-thirds of the nursing schools participating in the survey noted that faculty shortage is the primary reason for not accepting all qualified applicants into baccalaureate programs [1]. The consequence of a nursing shortage is nurses work longer hours under stressful conditions, which leads to nurses being more prone to making mistakes and medical errors. Subsequently, patient care suffers.

Schools of nursing are increasingly using hospital-based nurses to precept students during clinical rotations. These nurse preceptors extend the faculty at a time when a shortage of nursing faculty limits nursing school enrollment. Combined with initiatives already in place, such as using master's prepared nurses at the hospital as loaned faculty, compressing students' clinical rotations and assigning clinical rotations to off-shifts or, in less popular nursing units, using nurse preceptors as clinical faculty helps in two ways: it increases the number of available clinical nursing slots and it provides qualified clinical instructors. As little quantitative research on the effectiveness of using preceptors as clinical instructors early in a nursing program has been reported, this study looks at the question “Given baccalaureate students in their second medical-surgical class, do precepted students perform as academically well as traditionally prepared students?”

Clinical groups in Texas traditionally have a ratio of one master's prepared instructor to 10 students. The instructor's role is to monitor students in the clinical setting and instruct them in meeting their educational learning objectives. When class size is extended to increase enrollment, procuring a sufficient number of qualified clinical instructors is often difficult. That nurses qualified for teaching can make higher salaries working as a nurse in a hospital than as faculty in a university exacerbates the faculty shortage problem. Additionally, as the number of clinical slots dedicated to nursing students is limited, schools in the region compete with each other.

Nursing students today differ from students of past generations [2]. Students often demand accessible and timely information, and they want flexibility to meet their needs of working, studying, and raising a family. The younger students rely heavily on technology for learning, entertainment, and life scheduling. Learning experiences must be not only timely, but also relevant [3]. Students precepted one-on-one with registered nurses (RN) in hospital settings are more likely to find their needs met than is possible under a traditional group model, which permits less interaction between faculty and student, limits student opportunities for learning and skills practice, and provides an inaccurate view of the profession [4, 5]. 

According to the Texas Board of Nurse Examiners [6], master's prepared faculty can oversee the teaching activities of 12 RN preceptors, each of whom can supervise two undergraduate students. Using preceptors as clinical faculty alternatives more than doubles the number of students (*N* = 12 × 2 = 24) than can be placed in traditional clinical rotations (*N* = 10). The policy established by the Texas Board of Nursing states that a precepted student must be visited by a faculty member at least once a month. Faculty conducting the study rounded on precepted students at least twice a month since the students were early in their nursing program. 

Hospital-based clinical preceptors, as alternatives to clinical faculty, expect adequate support to function within the educator role [7]. According to Yonge and Myrick [8], preparation of preceptors includes teaching educational principles that help prepare the preceptors beyond their usual staff nurse orientation. Wilkes et al. [9] identified that continued support materials, beyond orientation, were essential to success. This support can be online and extended to faculty and students [10]. Using hand-held computers allows preceptors to obtain support at the bedside when time is at a premium and desktop services are not available [11]. The use of hand-held computers with internet access is an effective way for preceptors to obtain information, such as faculty contact information, school policies, and student clinical schedules, prepare anecdotal notes, and search for useful clinical information [12, 13].

Use of the precepting alternative also frees up additional nurses and hospital units for clinical training; nurses on smaller units not able to support a traditional group of 10 students could precept one-on-one and nurses working off-shifts can serve as preceptors [14]. Students participating in precepted groups find that they have more scheduling flexibility, greater opportunities for learning and practicing skills, and more relevant learning experiences [15].

To prepare the nurses for the preceptor role, the researchers developed a 14-module online preceptor education course. The module units, listed in Table 1, were designed to provide direction on teaching students as adult learners and to promote critical thinking in nursing students. The online training was offered through Blackboard. The project team, including faculty and hospital educators, attended the preceptors' presentations to offer technical training and support. Upon completion of the preceptor training, nurses received 9.6 continuing education units.

Having Blackboard access also gave the RN preceptors access to the students' course materials, including syllabi and lecture notes, which permitted the RN preceptors to provide clinical experiences that met the learning objectives and to keep pace with the students learning progression. As nurses traditionally work with new hire graduates and not students in their second semester of nursing education, it was important that preceptors knew that these students would not perform at the same level as a new graduate nurse.

Technology assisted in permitting continuous availability of faculty and in forming a communication net for students, preceptors, and faculty. Communication was via a dedicated web page, E-mail, cellular phones, traditional pagers, and handheld computers. Preceptors working at the bedside could communicate with nursing faculty in the office. Using the handheld computers, preceptors had a rapid means of access to relevant nursing and drug information while they were working with the students.

In addition, the research faculty and project staff developed a project-specific website to provide quick access to contact information, school policies, performance issue information, tips and topics, and links for emergencies and needle sticks. The website was a resource for preceptors and students whether the students were precepted during school hours or on off-shifts. Both preceptors and students could rapidly locate faculty contact information or receive technical support 24 hours a day. This was necessary as the precepted students worked the schedule their preceptor worked, whether this was a day, night, or weekend shift. E-mail group links permitted communication to the project staff and the control and experimental student groups. The availability of student performance information and school policies assisted preceptors to take appropriate action in addressing student attendance, dress and appearance, safety, and professionalism issues.

Using Blackboard to deliver the education course online permitted the RN preceptors to complete the training at their convenience; it also gave them access to additional learning resources. Since the students worked all shifts, discussion boards were set up to support communication and submission of assignments. Nursing faculty moderated the discussion postings and responded accordingly. Additionally, faculty and hospital educators periodically made rounds when students were scheduled to be with their preceptors to stay in contact, answer questions, and provide support. This also permitted faculty to assess student progress and preceptor effectiveness. The use of technology helped to decrease preceptor resistance in precepting students without the instructor being present and increased faculty assurance in the quality and relevance of the preceptors teaching.

The students had to complete a total of 96 hours in the clinical setting and were permitted to work between 8 and 24 hours per week. For faculty to keep track of student schedule and hours, students posted their clinical schedule at least 48 hours prior to working with their preceptor. Faculty held weekly post-clinical conferences with students at the school to answer questions and reinforce learning objectives. However, faculty were available to the students and preceptors by cellular phone twenty-four hours a day.

## 2. Methodology

The study used a quasi-experimental design where students (*N* = 69) were randomly assigned to a control group (traditional clinical group) or experimental group (precepted group). The subjects were in the second semester of their nursing education, and instruction that semester included pharmacology, gerontology, pathophysiology, and the second medical-surgical nursing course. Both experimental and control students volunteering to participate in the study submitted an informed consent form in accordance with the university's Institutional Review Board policy.

For experimental and control groups, the student's accumulative numerical course grade in medical-surgical course 1 (taken in the first semester) was used as an independent variable. The dependent variables were students' numerical grades on unit examinations given throughout the semester, comprehensive final examination grades, and accumulative numerical course grades in both the medical-surgical course 2 and the corequisite pharmacology course. Both experimental and control students took a standardized medical-surgical exam. Although scores earned on exams administered to measure what the student learned in the classroom does not have established reliability and validity for measuring clinical performance, issues surrounding clinical evaluation exist. Some of the identified issues pertinent to this study include the subjectivity of the evaluation especially when using “novice” clinical faculty. Novice faculty (bedside nurses serving as preceptors) may have limited formal education and experience in evaluation of students and often lack confidence in their ability to fairly evaluate students [16, 17].

Using a grading rubric, two faculty members on the research team reviewed the precepted student's nursing process papers at weeks 4, 8, and 12 and recorded these grades. To increase interrater reliability, the two researchers critiqued one student's paper prior to week 4 to standardize scoring. 

A clinical evaluation form was developed for preceptor use (Table 1 in supplementary material available online at doi:10.1155/2012/276506). After each 12-hour shift, the preceptor evaluated the student's clinical performance and faxed the form to the research team. Content covering the process of clinical evaluation was included within the online preceptor course. Faculty reviewed clinical evaluations after each shift worked by the student. If a student is consistently scoring 2 or less on the evaluation, the faculty meets with the preceptor to discuss the weaknesses of the student. Faculty would meet with the student as well to discuss strategies for improving their clinical performance. The final clinical evaluation was completed by the faculty and was based on data from the preceptor evaluations, the nursing process paper grades, and the faculty's impressions when rounding on the student in the hospital. 

### 2.1. Sample and Setting

The students were randomized into two groups, experimental or precepted group (*N* = 37) and control or traditional group (*N* = 32). The sample consisted of 6 male and 63 female students. The precepted students were assigned clinical rotations at two tertiary-level hospitals, where they worked one-on-one with a baccalaureate-prepared registered nurse (RN) on a medical or surgical unit or a surgical intermediate care unit. As per the request of the two hospitals, precepted students could not work on the days other school of nursing held their traditional clinical. This meant that there was one day a week in which the precepted students could not schedule a clinical day. Precepted students were not exposed to the traditional students or their faculty when working. The control students were mixed within the traditional training groups of 10 students, and these groups were assigned to medical-surgical floors at various local hospitals.

Hospital educators recruited the preceptors. The criteria for eligibility were that the RNs (1) have at least one year experience as a registered nurse; (2) have a recent satisfactory annual evaluation by their nurse manager; (3) have a current BCLS and nursing license; (4) completed the online preceptor education course; (5) graduated from a baccalaureate nursing program. Inclusion criteria for students were that they were in their second semester of nursing school and had not previously withdrawn from or failed a class.

### 2.2. Data Analysis

The students' final numerical course grade from the first semester medical-surgical course was analyzed using an independent sample *t*-test to determine whether the first semester grade needed to be used as a co-variant. However, this test showed no significant difference between the experimental and control designated groups. Student 2nd semester medical-surgical examination scores (4 units and 1 comprehensive final) were analyzed using a mixed model approach for repeated measures ANOVA. Final numerical grades from their pharmacology course and a standardized specialty medical-surgical examination (HESI) were analyzed using independent sample *t*-tests.

## 3. Results

No significant differences between the experimental and control groups on any measurement were found. For discussion purposes, an implication is that precepted students did as academically well as students in the control group. Likewise, this could be stated, as precepted students do no better than traditional students academically despite the one-on-one clinical treatment. Given this, the study supports using hospital-based RN nurses as clinical preceptors. Using RNs as preceptors not only provides much needed clinical faculty but also frees up clinical slots that previously have not been available.

### 3.1. Analysis of the Medical-Surgical Course Grades

The four unit examination grades and the final examination grade were analyzed using a mix model approach for repeated measures ANOVA. There was no statistically significant difference between the precepted and control groups (*F* = .936, df = 63, *P* = .449). Mean grade scores for the examinations are presented in Table 2. 

An independent sample *t* test was computed to determine if there was a significant difference between precepted and control groups in academic performance as demonstrated by the final course grade students received in their second semester medical-surgical course. Students in the traditional clinical group had a mean of 83.7 (SD = 5.9), and the precepted students had a mean of 83.5 (SD = 4.6). There was no statistical difference between the two groups (*t* = .118, df = 67, *P* = .906).

### 3.2. HESI Medical-Surgical Specialty Exam

As second semester students take the Health Education Services Inc. (HESI, now owned by Elsevier) medical-surgical specialty exam at the end of the semester, this mean score was considered in the analysis. The mean standard score for the traditional students was 784.19 (SD = 182.5) as compared to a mean standard score of 807.76 (SD = 136.7) for the precepted students. However, there was no statistical difference between the two groups (*t* = −.612, df = 67, *P* = .543).

### 3.3. Pharmacology Final Course Grade

Students in the traditional clinical groups usually prepared medication cards the day before each clinical session. As students in the precepted group did not know which patients their preceptor would have until report, they had to take a different approach. They reviewed each medication and presented the information to their preceptor before administering the medication. In analyzing pharmacology final course grades, there was no statistical difference between the two groups (*t* = −.786, df = 67, *P* = 434). The students in the control group had a mean grade of 89.1 (SD = 3.8), and the precepted group had a mean of 88.3 (SD = 4.8).

### 3.4. Clinical Evaluations

On average, the students in the precepted group received ratings of 3–5 for the clinical competencies listed on the clinical evaluation form. As expected, some preceptors were more confident in their role as clinical evaluator. Confident preceptors not only rated the clinical performance, but also provided anecdotal information to justify the given ratings. Preceptors did note that the clinical evaluation process was an added responsibility to their busy clinical day.

### 3.5. Nursing Process Papers

Unlike students in the traditional group, precepted students arrived for their shift with no preparatory work in place. The student and their preceptor selected one patient as the student's primary patient for that shift. Although the precepted student was responsible for obtaining the same information as that of a traditional student, the precepted students did this on shift and completed the nursing process papers retrospectively. The faculty found that the quality of the nursing process papers were similar between the precepted and the traditional groups. Students in both groups struggled with parts of the nursing diagnosis (“related to” statement) and setting measurable expected outcomes. Precepted students demonstrated a better comprehension of the importance of replanning. Another difference between the two groups was timeliness. The precepted students, instructed to turn in their paperwork within a week of completing the shift, tended to fall behind, allowing the paperwork to accumulate.

### 3.6. Challenges

The research team confronted several challenges during the two-year study. One major challenge was merging the technology of two different institutions via Internet. The information technology (IT) member of the research team worked closely with the two hospitals that participated in the study. The IT personnel from the two hospitals and the university had to breech the “firewall” that existed between institutions. IT teams on both sides were involved and were able to accomplish this task. Once the two systems were connected, the IT member of the research team met with preceptors one-on-one to help them navigate the Blackboard site within the university. The IT personnel discovered that many nurses were not technologically perceptive. The IT member held mini tutorials to help the preceptors learn how to use their institution's intranet, access the university's Blackboard site, and navigate the preceptor course. This challenge was not anticipated by the research team and did require additional time not planned in the original proposal.

 Overall, the experiences with the preceptors were positive. Most preceptors had served in the role in the past, orienting new hires on their respective units. One occasion arose where a preceptor placed the student in a dangerous situation (administering medications without being present). The student reported the incident, and working with the institution, the preceptor was replaced with another nurse.

Another challenge was the evaluation process by the preceptors. Although many of the preceptors have mentored new nurses on their units, they had not participated in a formal evaluation process. Some preceptors would circle all the same number on the Likert scale clinical performance evaluation and would not provide any commentary. The members of the research team would meet with preceptors and ask for a verbal evaluation of the student's performance. Questions asked included an assessment of the student's ability to perform a focused review of systems and physical exam, knowledge of medications and their administration, and the potential to use the nursing process to plan appropriate care for the patient, for example. Meeting with preceptors frequently provided more insights into the clinical performance of the student. Reviewing the nursing process papers also provided insights as to how the student was performing in the clinical setting.

## 4. Discussion

In the last decade, we have seen an increase in the number of applicants to nursing programs, a decline in the number of nurses in the workforce, and a decline in the number of nurses who pursue a career in academia. With the nursing shortage, demands are made on schools to increase enrollment; however, with the shortage of nursing faculty, this demand has been difficult to meet. As a barrier to increasing enrollment has been clinical availability [5], a solution is using nurse preceptors to extend faculty in the clinical setting.

This project examined the use of preceptors, supported by training and technology, to facilitate the clinical experience of students in their second semester medical-surgical course. Precepting students are not a new concept within nursing; however, a preceptor support model for students early in their nursing education has not been fully studied [14]. The purpose of the study was to determine whether students who were precepted performed as well as those in a traditional clinical group. As there was no significant difference in performance on grades and HESI scores, the premise is upheld. The results suggest that from an academic perspective, providing clinical education when using qualified and trained preceptors did not interfere with the student's ability to master the course content.

The quality of the nursing process papers produced by students was deemed to be equal between the two groups. Students in both groups were provided feedback on their papers and were asked in equal proportion to resubmit work. The quality of the medication information sheets was found to be equivalent. As the main problem encountered was the timeliness of the submission of the paperwork, a solution would be to design a mechanism within the computer-scheduling program that locked out students from posting their schedule until all nursing process paperwork was completed.

## Supplementary Material

Clinical evaluation form developed for preceptors to evaluate the students' progress after each clinical day was completed.

## Figures and Tables

**Table 1:** Online Modules Available to Nurse Preceptors.

Module No.	Module title
1	Nurses and the educational process
2	Preceptorship and nursing education
3	Applying learning theories to teaching
4	Assessing learning styles
5	Learning contracts
6	Motivation to learn
7	Critical thinking
8	Communicating with students
9	The impact of technology on education
10	Ethical and legal issues in clinical nursing education
11	Clinical evaluation of students
12	Evidence-based practice
13	Cultural competency and health care
14	Educating students with special needs

**Table 2:** Mean Scores for 2nd semester BSN student medical-surgical course.

Examination	Group	Mean	SD
1	Control	88.7	6.3
	Experimental	88.4	5.5
2	Control	87.3	7.9
	Experimental	87.6	7.3
3	Control	84.9	5.5
	Experimental	86.1	5.5
4	Control	83.0	8.5
	Experimental	81.1	9.0
Comprehensive	Control	78.1	8.1
	Experimental	77.9	6.8
