# Supplementary material for: A Comparison of a Traditional Clinical Experience to a Precepted Clinical Experience for Baccalaureate-Seeking Nursing Students in Their Second Semester
Source: Nurs Res Pract. 2012 Apr 22;2012:276506. doi: 10.1155/2012/276506 (PMC3346989; doi:10.1155/2012/276506)
Supplement: Supplementary file 1 — Clinical evaluation form developed for preceptors to evaluate the students' progress after each clinical day was completed. [file 276506.f1.pdf]

Table 1

Preceptor Evaluation of Student

Student Name:____

Date ________________________ Number of Hours:____________

Preceptor:_______________________ Hospital:__________________

Please complete this form after each clinical session with the student. If you answer no to questions 1-4 or rate 1-2 on questions 5-26, please notify the instructor.

	No	Yes	
1. Was on time	1	2	If no, Arrival Time __________
2. Was dressed in UT student uniform	1	2	
3. Maintains confidentiality of patient	1	2	
4. Safely administers medications	1	2	

1-Unsatisfactory 2-Needs Improvement 3-Average 4-Good 5 Excellent

Member of a Profession:

5. Communicates in a goal-directed manner 1 2 3 4 5
6. Demonstrates ability to think critically 1 2 3 4 5
7. Seeks constructive feedback regarding practice 1 2 3 4 5
8. Seeks knowledge/skill appropriate to course 1 2 3 4 5

Provider of Care

Assessment:

9. Accurately interprets the meaning of lab values 1 2 3 4 5
10. Obtains significant data from patient/family/records 1 2 3 4 5
11. Is sensitive to socio-cultural aspects of patient/family 1 2 3 4 5

Diagnoses:

12. Determines nursing diagnoses appropriate to patient data 1 2 3 4 5
13. Recognizes priority care problems 1 2 3 4 5
14. Discusses pathology related to problem statement 1 2 3 4 5

Plan:

15. Goal criteria are realistic 1 2 3 4 5

Implementation:

16. Nursing interventions are safely completed 1 2 3 4 5
17. Interventions are individualized to patient/family 1 2 3 4 5
18. Supports interventions with scientific principles 1 2 3 4 5
19. Demonstrates initiative in performing patient care 1 2 3 4 5
20. Demonstrates basic knowledge of medications 1 2 3 4 5

Evaluation:

21. Determines if goal achieved 1 2 3 4 5
22. Modifies nursing interventions appropriately, if needed 1 2 3 4 5

Coordinator of Care

23. Safely performs patient care 1 2 3 4 5
24. Reports changes in patient conditions to preceptor/staff 1 2 3 4 5
25. Establishes a collaborative relationship with others 1 2 3 4 5
26. Charting meets the guidelines of the institution 1 2 3 4 5

Comments____

____

Preceptor's signature____
